# Correction: Ravin et al. Two New Species of Filamentous Sulfur Bacteria of the Genus *Thiothrix*, *Thiothrix winogradskyi* sp. nov. and ‘*Candidatus* Thiothrix sulfatifontis’ sp. nov. *Microorganisms* 2022, *10*, 1300

**DOI:** 10.3390/microorganisms10081665

**Published:** 2022-08-18

**Authors:** Nikolai V. Ravin, Simona Rossetti, Alexey V. Beletsky, Vitaly V. Kadnikov, Tatyana S. Rudenko, Dmitry D. Smolyakov, Marina I. Moskvitina, Maria V. Gureeva, Andrey V. Mardanov, Margarita Yu. Grabovich

**Affiliations:** 1 Institute of Bioengineering, Research Center of Biotechnology of the Russian Academy of Sciences, 119071 Moscow, Russia; 2 Water Research Institute, IRSA-CNR, Monterotondo, 00185 Rome, Italy; 3 Department of Biochemistry and Cell Physiology, Voronezh State University, Universitetskaya pl., 1, 394018 Voronezh, Russia

The authors wish to make the following corrections to this paper [1]:

There are mistakes regarding the possibility for growth on several substrates shown in Table 1 and in the table legend because some lines were changed when formatting the table.

The correct version is as follows:

**Table 1:** Differentiating characteristics between phylogenetically related species from the genus *Thiothrix* (Clade I). All strains utilize acetate, lactate and pyruvate. Glyoxylate, glycolate, benzoate, salicylate, methanol, isopropanol (propan-2-ol), glycerol, lactose, D-galactose, D-(+)-glucose, D-mannose, serine, lysine, tryptophan, methionine, tyrosine, ornithine, glutamine and alanine do not support the growth of all strains. All strains are capable of chemolitoautotrophic growth with thiosulfate and bisulfide. All strains are capable of respiration of nitrate to nitrite. No strains hydrolyze gelatin or starch. All strains are catalase-negative. None of the strains grow at 3% NaCl. *Thiothrix litoralis* AS^T^ is able to survive with up to 3% NaCl in the medium. The major fatty acids for all strains are C_16:1_^ω7^, C_16:0_, C_18:1_^ω7^. Data are from [26,27].

Characteristic	*Thiothrix* sp. CT3	*T*. *litoralis* AS^T^	*T*. *lacustris* BL^T^	*T*. *fructosivorans* I	*T*. *caldifontis* G1^T^	*T*. *subterranea* Ku-5^T^
Natural habitat	activated sludge	seashore of the White Sea	sulfide spring	activated sludge	sulfide spring	sulfide-containing waters from a coal mine
Cell size (µm)	0.8–2.0 × 4.3–6.7	0.8–2.2 × 4.3–6.4	0.9–2.3 × 4.4–6.3	1.0–1.7 × 4.9–10.0	0.9–2.2 × 3.2–6.5	1.19–1.8 × 4.0–6.3
Optimum (range) pH for growth	7.6 (7.0–8.0)	7.4–7.5 (6.7–8.0)	7.0 (6.2–8.2)	7.6–8.0 (6.7–8.0)	8.0 (7.0–8.6)	7.4–7.5 (6.8–8.0)
Optimum (range) temperature for growth (°C)	20–24 (10–30)	20–22 (4–28)	24 (5–32)	25–27 (5–32)	25 (7–37)	20–22 (4–28)
Organic substrates utilized for growth						
Organic acids:						
Malate	−	−	−	+	−	−
Oxalate	−	−	+	+	−	+
Oxaloacetate	−	−	+	+	+	+
Citrate	−	−	+	−	−	−
Isocitrate	−	−	+	+	−	−
2-oxoglutarate	−	−	+	−	−	−
Formate	−	−	−	+	−	−
Aconitate	−	−	+	−	−	+
Malonate	−	+	+	−	−	+
Succinate	+	+	+	+	+	−
Alcohols:						
Inositol	−	−	−	−	−	+
Ethanol	−	−	−	−	−	+
Butanol	−	−	−	−	−	+
Isobutanol	−	−	−	−	−	+
Mannitol	−	−	−	−	−	+
Sorbitol	−	−	−	−	−	+
Carbohydrates:						
L-Arabinose	−	−	−	+	−	+
D-Xylose	−	−	−	+	−	−
D-Fructose	−	+	−	+	−	+
L-Rhamnose	−	−	−	−	−	+
L-Sorbose	−	−	−	−	−	+
Sucrose	−	−	−	+	−	−
Maltose	−	+	−	+	−	+
Trehalose	−	+	−	−	−	−
Raffinose	−	−	−	+	−	+
Amino acids:						
Isoleucine	−	+	−	−	+	+
Leucine	−	+	−	−	+	+
Proline	−	−	−	−	−	+
Cystein	−	−	+	−	−	−
Asparagine	−	+	+	−	−	−
Phenylalanine	−	−	−	−	−	+
Aspartate	−	+	+	−	+	−
Glutamate	−	+	+	−	−	−
Histidine	−	−	−	−	−	+
Complex media:						
Peptone	−	+	−	−	−	+
Yeast extract	−	+	−	−	−	−
Diazotrophy	−	+	−	−	+	+
Major fatty acids:						
C_16:1_^ω7^, C_16:0_, C_18:1_^ω7^	+	+	+	+	+	+

The authors apologize for any inconvenience caused and state that the scientific conclusions are unaffected. The original publication has also been updated.
