# Supplementary material for: Two New Species of Filamentous Sulfur Bacteria of the Genus Thiothrix, Thiothrix winogradskyi sp. nov. and ‘Candidatus Thiothrix sulfatifontis’ sp. nov
Source: Microorganisms. 2022 Jun 27;10(7):1300. doi: 10.3390/microorganisms10071300 (PMC9319827; doi:10.3390/microorganisms10071300)
Supplement: Supplementary file 1 [file microorganisms-10-01300-s001.zip › microorganisms-1772905-supplementary.pdf]

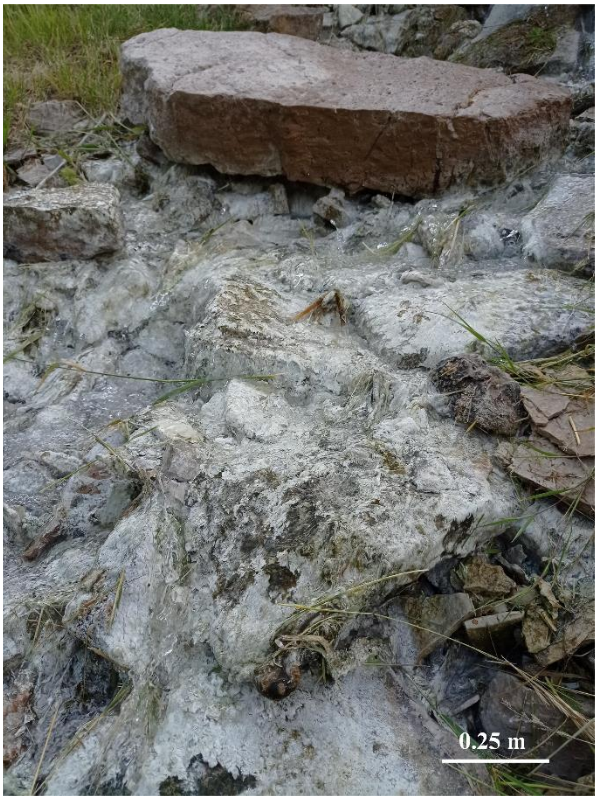

Figure S1. Bacterial mats of *Thiothrix* in the hydrogen sulfide spring "Serovodorodny" in Tatarstan, Russia.

Genome	AS^{T}	BL^{T}	Q^{T}	G1^{T}	**CT3^{T}**	**KT**	Ku-5^{T}	A1^{T}	A52	RT	JP2^{T}	SSD2
AS^{T}	100	56.4	28.6	28.6	27.6	28.8	28.5	28.2	27.4	25.3	24.7	22.6
BL^{T}	56.4	100	28.8	28	27.9	27.8	27.7	27.8	27.7	22.4	24.9	23.9
Q^{T}	28.6	28.8	100	32.9	32.6	33.5	34.7	35.1	38.9	30.5	23.7	22.3
G1^{T}	28.6	28	32.9	100	36	36.4	38.5	37.3	35	22.7	30.2	23.8
**CT3^{T}**	27.6	27.9	32.6	36	100	33.3	40.1	34.2	31.9	22.3	30.5	23.7
**KT**	28.8	27.8	33.5	36.4	33.3	100	48.7	33.8	33.8	27.7	23.6	22.9
Ku-5^{T}	28.5	27.7	34.7	38.5	40.1	48.7	100	36.7	35.9	27.9	23.6	22.4
A1^{T}	28.2	27.8	35.1	37.3	34.2	33.8	36.7	100	46.9	30.5	23.4	23
A52	27.4	27.7	38.9	35	31.9	33.8	35.9	46.9	100	21.3	24.6	32.3
RT	25.3	22.4	30.5	22.7	22.3	27.7	27.9	30.5	21.3	100	25.6	24.9
JP2^{T}	24.7	24.9	23.7	30.2	30.5	23.6	23.6	23.4	24.6	25.6	100	27.3
SSD2	22.6	23.9	22.3	23.8	23.7	22.9	22.4	23	32.3	24.9	27.3	100

dDDH, %
100
36
22

Figure S2. dDDH pairwise values for *Thiothrix* genomes. *T. lacustris* BL^{T}, (GCF_000621325.1); *T. litoralis* AS^{T} (GCF_017901135.1); *T. subterranea* Ku-5^{T} (GCF_016772315.1); MAG of *Thiothrix* sp. KT (GCA_022828425.1); *T. caldifontis* G1^{T} (GCF_900107695.1); *Thiothrix* sp. CT3 (GCA_021650935.1); *T. fructosivorans* Q^{T} (GCA_017349355.1); *T. unzii* A1^{T} (GCA_017901175.1); MAG of *Thiothrix* sp. A2; MAG of *Thiothrix* sp. A52 (GCF_017901155.1); '*Ca.* Thiothrix moscowensis' RT (GCA_016292235.1); *T. nivea* JP2^{T} (GCF_000260135.1); '*Ca*. Thiothrix singaporensis' SSD2 (GCA_013693955.1).

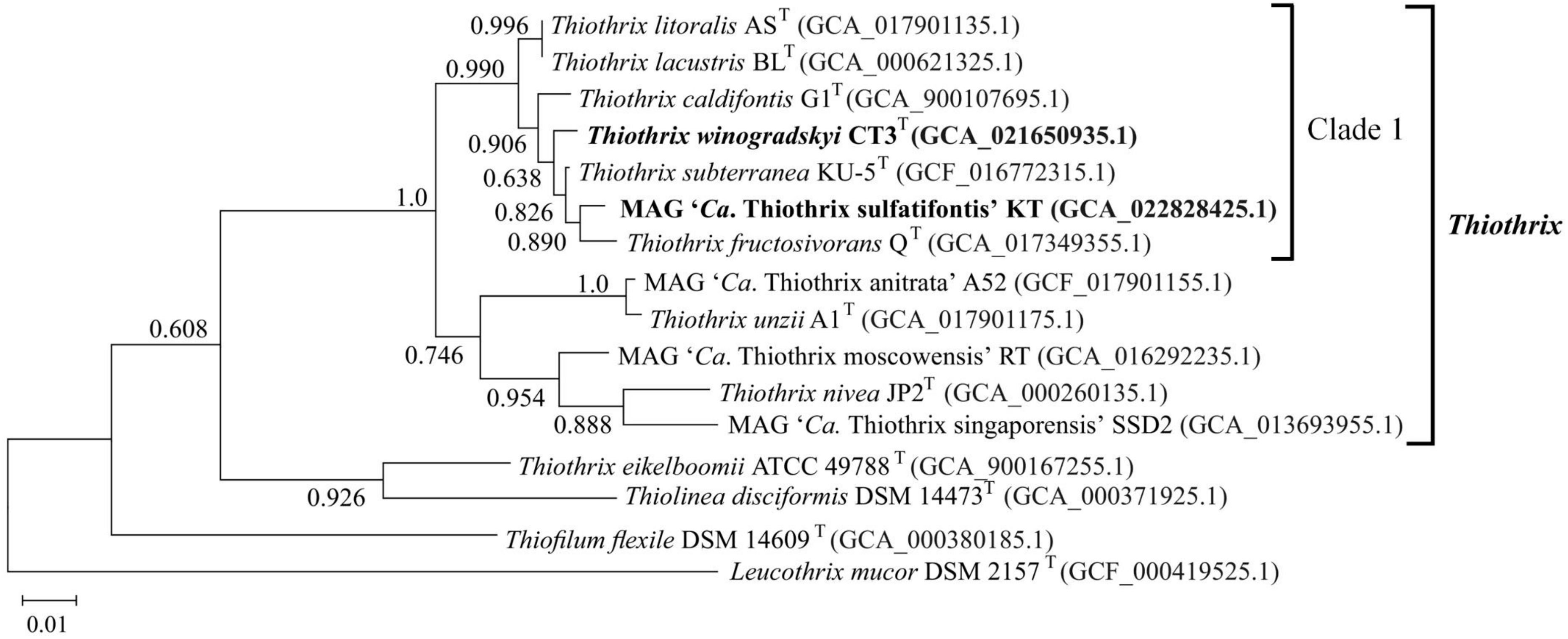

Figure S3. 16S rRNA gene-based maximum-likelihood phylogenetic tree of type strains of *Thiothrix* species. The 16S rRNA gene sequences extracted from genomes (assembly accession numbers are listed after the genome name) were aligned against the SILVA database using SINA v.1.2.11. The alignment had 1535 positions. The best evolutionary model, HKY+G+I (HKY substitution model, estimated proportion of invariable sites, estimated gamma shape parameter), was defined by jModelTest v.2.1.10 with the Bayesian Information Criterion. The maximum likelihood tree was constructed using PhyML v.3.3 with 500 bootstrap replicates; the Log-likelihood of the tree was -5038.18080. The internal branching support levels are specified at the nodes. The genome of *Leucothrix mucor* DSM 2157^{T} was used for tree rooting. Scale, 0.01 substitutions per nucleotide position. Note that *Thiothrix eikelboomii* actually should belong to the genus *Thiolinea* but cannot be reclassified because of its absence in two international collections [3].

Table S1. Genes encoding enzymes of metabolic pathways in '*Candidatus* Thiothrix sulfatifontis' KT and *Thiothrix winogradskyi* CT3^{T}.

Gene	Predicted function	KT	CT3
		RefSeq (GeneBank) locus_tag	RefSeq (GeneBank) locus_tag
**Sulfur metabolism**			
sqrA	Sulfide quinone oxidoreductase SqrA (EC 1.8.5.4)	L3K52_05845	L2Y54_RS07100
sqrF	Sulfide quinone oxidoreductase SqrF (EC 1.8.5.4)	L3K52_13295	L2Y54_RS12370<br>L2Y54_RS12360
fccB	Sulfide dehydrogenase (flavocytochrome c) flavoprotein chain	L3K52_05350<br>L3K52_18375<br>L3K52_10900	L2Y54_RS06570<br>L2Y54_RS06620<br>L2Y54_RS21270<br>L2Y54_RS15945
fccA	cytochrome c subunit of flavocytochrome c sulfide dehydrogenase	L3K52_05185	L2Y54_RS06390
soxA	sulfur oxidation c-type cytochrome SoxA (EC 1.8.2.3)	L3K52_01425	L2Y54_RS05655<br>L2Y54_RS10175<br>L2Y54_RS11445
soxZ	thiosulfate oxidation carrier complex protein SoxZ (EC 1.8.2.6)	L3K52_01420<br>L3K52_01435	L2Y54_RS05660<br>L2Y54_RS10180
soxY	thiosulfate oxidation carrier protein SoxY (EC 1.8.2.6)	L3K52_01415<br>L3K52_01430	L2Y54_RS05665<br>L2Y54_RS10185
soxB	thiosulfohydrolase SoxB (EC 3.1.6.20)	L3K52_01470	L2Y54_RS05615
soxX	sulfur oxidation c-type cytochrome SoxX (EC 1.8.2.3)	L3K52_01410	L2Y54_RS05670<br>L2Y54_RS10170<br>L2Y54_RS11440
soeA	sulfite dehydrogenase (quinone) subunit SoeA (EC 1.8.5.6)	L3K52_02785	L2Y54_RS04105
soeB	sulfite dehydrogenase (quinone) subunit SoeB (EC 1.8.5.6)	L3K52_02780	L2Y54_RS04110
soeC	sulfite dehydrogenase (quinone) subunit SoeC (EC 1.8.5.6)	L3K52_02775	L2Y54_RS04115

aprA	adenylylsulfate reductase, subunit A (EC 1.8.4.10)	L3K52_04580	L2Y54_RS05790
aprB	adenylylsulfate reductase, subunit B (EC 1.8.4.10)	L3K52_04575	L2Y54_RS05785
sat	sulfate adenylyltransferase (EC 2.7.7.4)	L3K52_04570	L2Y54_RS05780
dsrA	Dissimilatory sulfite reductase, alpha subunit (EC 1.8.99.5)	L3K52_08855	L2Y54_RS11490
dsrB	Dissimilatory sulfite reductase, beta subunit (EC 1.8.99.5)	L3K52_08860	L2Y54_RS11495
dsrE	tRNA 2-thiouridine synthesizing protein D (EC 2.8.1.-)	L3K52_08865	L2Y54_RS11500
dsrF	tRNA 2-thiouridine synthesizing protein C (EC 2.8.1.-)	L3K52_08870	L2Y54_RS11505
dsrH	tRNA 2-thiouridine synthesizing protein B (EC 2.8.1.-)	L3K52_08875	L2Y54_RS11510
dsrC	tRNA 2-thiouridine synthesizing protein E (EC 2.8.1.-)	L3K52_08835<br>L3K52_08880	L2Y54_RS11470<br>L2Y54_RS11515
dsrM	sulfite reduction-associated complex DsrMKJOP protein DsrM	L3K52_08885	L2Y54_RS11520
dsrK	sulfite reduction-associated complex DsrMKJOP protein DsrK	L3K52_08890	L2Y54_RS11525
dsrL	glutamate synthase (NADPH) small chain dsrL/ intracellular sulfur oxidation protein DsrL	L3K52_08895	L2Y54_RS11530
dsrJ	Sulfite reduction-associated complex DsrMKJOP multiheme protein DsrJ	L3K52_08900	L2Y54_RS11535
dsrO	sulfite reduction-associated complex	L3K52_08905	L2Y54_RS11540

	DsrMKJOP iron-sulfur protein DsrO		
dsrP	sulfite reduction-associated complex DsrMKJOP protein DsrP	L3K52_08910	L2Y54_RS11545
dsrN	Cobyrinic acid A,C-diamide synthase DsrN (EC 6.3.5.11)	L3K52_11740	L2Y54_RS14880
dsrR	protein involved in sulfur oxidation DsrR	L3K52_11745	L2Y54_RS14875
dsrS	protein involved in sulfur oxidation DsrS	L3K52_11645	L2Y54_RS14955
**Nitrogen metabolism**			
narG	nitrate reductase alpha subunit (EC 1.7.99.4)	-	L2Y54_RS20190
narH	nitrate reductase beta subunit (EC 1.7.5.1)	-	L2Y54_RS20185
narI	nitrate reductase gamma subunit (EC 1.7.5.1)	-	L2Y54_RS20175
narJ	nitrate reductase delta subunit (EC 1.7.5.1)	-	L2Y54_RS20180
nirS	nitrite reductase (NO-forming) (EC 1.7.2.1)	-	L2Y54_RS20045
cnorB	nitric oxide reductase subunit B (EC 1.7.2.5)	-	L2Y54_RS20040
cnorC	nitric oxide reductase subunit C (EC 1.7.2.5)	-	L2Y54_RS20035
nirB	nitrite reductase (NADH) large subunit (EC 1.7.1.1)	L3K52_06745	L2Y54_RS07995
nirD	nitrite reductase (NADH) small subunit (EC 1.7.1.1)	L3K52_06750	L2Y54_RS08000
nifA	Nif-specific regulatory protein NifA	L3K52_17530	-
nifS	cysteine desulfurase NifS (EC 2.8.1.7)	L3K52_17560	-
nifU	nitrogen fixation protein NifU and related proteins	L3K52_17565	-
nifB	nitrogen fixation protein NifB	L3K52_17615	-

nifX	nitrogen fixation protein NifX	-	-
nifX2	nitrogen fixation protein NifX2	-	-
nifB2	nitrogen fixation protein NifB2	L3K52_12300	-
nifY	nitrogen fixation-related protein, gamma subunit	-	-
nifE	nitrogenase molybdenum-cofactor synthesis protein NifE (EC 1.18.6.1)	L3K52_08140	-
nifN	nitrogenase molybdenum-iron protein NifN (EC 1.18.6.1.)	L3K52_08145	-
nifQ	nitrogen fixation protein NifQ	L3K52_17585	-
nifV	homocitrate synthase NifV (EC 2.3.3.14)	L3K52_08110	-
nifW	nitrogen fixation protein NifW	L3K52_08070	-
nifM	nitrogen fixation protein NifM	L3K52_08060	-
nifH	nitrogenase iron protein NifH (EC 1.18.6.1)	-	-
nifD	nitrogenase molybdenum-iron protein alpha chain (EC 1.18.6.1)	-	-
nifK	nitrogenase molybdenum-iron protein beta chain (EC 1.18.6.1)	-	-
nifZ	nitrogen fixation protein NifZ	L3K52_08065	-
nifT	nitrogen fixation protein NifT	-	-
nifO	NifO	L3K52_17600	-
nasA	assimilatory nitrate reductase catalytic subunit	L3K52_06755	L2Y54_RS08005
**Phosphorus metabolism**			
phoU	phosphate transport system protein PhoU	L3K52_14400	L2Y54_RS13770<br>L2Y54_RS13790

phoR	Phosphate regulon sensor protein PhoR (EC 2.7.13.3)	L3K52_14410	L2Y54_RS13780
phoB	Phosphate regulon transcriptional regulatory protein PhoB (EC 2.7.13.3)	L3K52_14170<br>L3K52_14405	L2Y54_RS12815<br>L2Y54_RS13775
pstS	phosphate transport system permease protein PstS	L3K52_00885<br>L3K52_11775<br>L3K52_14215<br>L3K52_14360	L2Y54_RS10225<br>L2Y54_RS13730<br>L2Y54_RS14835
pstA	phosphate transport system permease protein PstA (EC 7.3.2.1)	L3K52_14390	L2Y54_RS13760
pstC	phosphate transport system permease protein PstC (EC 7.3.2.1)	L3K52_14385	L2Y54_RS13755
pstB	pstA; phosphate transport system permease protein PstB (EC 7.3.2.1)	L3K52_14395	L2Y54_RS13765
ppk1	polyphosphate kinase (EC 2.7.4.1)	L3K52_16915	L2Y54_RS19505
epp	Exopolyphosphatase (EC 3.6.1.11)	L3K52_01755	L2Y54_RS05070
**Embden-Meyerhof (EM) pathway and Calvin-Benson-Bassham cycle**			
glk	Glucokinase (EC 2.7.1.2)	L3K52_02195	L2Y54_RS04615
pgi	glucose-6-phosphate isomerase (EC 5.3.1.9)	L3K52_02200	L2Y54_RS04610
pfk	6-phosphofructokinase (EC 2.7.1.11)	L3K52_04600	L2Y54_RS05810
fbaA	fructose-bisphosphate aldolase class II (EC 4.1.2.13)	L3K52_04730	L2Y54_RS05940
tpiA	triosephosphate isomerase (EC 5.3.1.1)	L3K52_08945	L2Y54_RS11580
gapA	NAD-dependent glyceraldehyde-3-phosphate dehydrogenase (EC 1.2.1.12)	L3K52_04880<br>L3K52_04715	L2Y54_RS01965<br>L2Y54_RS10245<br>L2Y54_RS05925
pgk	Phosphoglycerate kinase (EC 2.7.2.3)	L3K52_04720	L2Y54_RS05930

gpmI	2,3-bisphosphoglycerate-independent phosphoglycerate mutase (EC 5.4.2.1)	L3K52_04765	L2Y54_RS02070
eno	Enolase (EC 4.2.1.11)	L3K52_10575	L2Y54_RS12835
pyk	Pyruvate kinase (EC 2.7.1.40)	L3K52_14355<br>L3K52_04725	L2Y54_RS13725<br>L2Y54_RS05935
porA	pyruvate ferredoxin oxidoreductase alpha subunit [EC:1.2.7.1]	L3K52_02800	L2Y54_RS04055
porB	pyruvate ferredoxin oxidoreductase beta subunit [EC:1.2.7.1]	L3K52_02805	L2Y54_RS04050
porC, porG	pyruvate ferredoxin oxidoreductase gamma subunit [EC:1.2.7.1]	L3K52_02795	L2Y54_RS04060
aceE	Pyruvate dehydrogenase E1 component (EC 1.2.4.1)	L3K52_14985	L2Y54_RS17170<br>L2Y54_RS04245
pdhC	Dihydrolipoamide acetyltransferase component of pyruvate dehydrogenase complex (EC 2.3.1.12)	L3K52_14980	L2Y54_RS17165<br>L2Y54_RS04240
pdhD	Dihydrolipoamide dehydrogenase of pyruvate dehydrogenase complex (EC 1.8.1.4)	L3K52_14975	L2Y54_RS17160
		L3K52_10195	L2Y54_RS04235
rpiA	Ribose-5-phosphate isomerase A (EC 5.3.1.6)	L3K52_05760	L2Y54_RS06975
rpe	Ribulose-phosphate 3-epimerase (EC 5.1.3.1)	L3K52_15085	L2Y54_RS17270
tktA	Transketolse (EC 2.2.1.1)	L3K52_04710	L2Y54_RS05920
fbp	Fructose-1,6-bisphosphatase, type I (EC 3.1.3.11)	L3K52_03050	L2Y54_RS03730

rbcL	Ribulose bisphosphate carboxylase large chain (EC 4.1.1.39)	L3K52_12120<br>L3K52_00755<br>L3K52_15820	L2Y54_RS00875<br>L2Y54_RS01010
rbcS	Ribulose bisphosphate carboxylase small chain (EC 4.1.1.39)	L3K52_00760<br>L3K52_15825	L2Y54_RS01015
prkB	Phosphoribulokinase (EC 2.7.1.19)	L3K52_10935<br>L3K52_04410	L2Y54_RS02325<br>L2Y54_RS15920
**Tricarboxylic acid (TCA) cycle, glyoxylate cycle and a way to anabolism from TCA**			
gltA	Citrate synthase (si) (EC 2.3.3.1)	L3K52_10210	L2Y54_RS16985
acnB	Aconitate hydratase 2 (EC 4.2.1.3)	L3K52_10215	L2Y54_RS16980
idh	Isocitrate dehydrogenase (NADP) (EC 1.1.1.42)	L3K52_12060	L2Y54_RS14605<br>L2Y54_RS18995<br>L2Y54_RS06290
sucA	2-oxoglutarate dehydrogenase E1 component (EC 1.2.4.2)	L3K52_13190<br>L3K52_11440	L2Y54_RS18995<br>L2Y54_RS06290
sucB	2-oxoglutarate dehydrogenase E2 complex (EC 2.3.1.61)	L3K52_10190	L2Y54_RS12490
sucD	Succinyl-CoA ligase (ADP-forming) alpha chain (EC 6.2.1.5)	L3K52_10205	L2Y54_RS13255
sucC	Succinyl-CoA ligase (ADP-forming) beta chain (EC 6.2.1.5)	L3K52_10200	L2Y54_RS16995
sdhA	Succinate dehydrogenase flavoprotein subunit (EC 1.3.99.1)	L3K52_13210	L2Y54_RS12470
sdhB	Succinate dehydrogenase iron-sulfur protein (EC 1.3.99.1)	L3K52_13215	L2Y54_RS12465
sdhC	Succinate dehydrogenase cytochrome b-556 subunit (EC 1.3.99.1)	L3K52_13200	L2Y54_RS12480
sdhD	Succinate dehydrogenase hydrophobic	L3K52_13205	L2Y54_RS10030

	membrane anchor protein (EC 1.3.5.1)		
fumA	Fumarate hydratase class I, aerobic (EC 4.2.1.2)	L3K52_13100	L2Y54_RS12610
mqo	Malate:quinone oxidoreductase (EC 1.1.5.4)	L3K52_16865	L2Y54_RS19455
pckA	Phosphoenolpyruvate carboxykinase (ATP) (EC 4.1.1.49)	L3K52_10325	L2Y54_RS16795
aspB	Aspartate aminotransferase (EC 2.6.1.1)	L3K52_11185<br>L3K52_15490<br>L3K52_15450	L2Y54_RS15595<br>L2Y54_RS17995
gltB	Glutamate synthase (NADPH) large chain (EC 1.4.1.13)	L3K52_15405	L2Y54_RS17900
gltD	Glutamate synthase (NADPH) small chain (EC 1.4.1.13)	L3K52_15410	L2Y54_RS17905
aceA	Isocitrate lyase (EC 4.1.3.1)	L3K52_16860	L2Y54_RS19450
aceB	Malate synthase G (EC 2.3.3.9)	L3K52_01335	L2Y54_RS06200
maeB	NADP-dependent malic enzyme (EC 1.1.1.40)	L3K52_01365	L2Y54_RS06165
ppc	phosphoenolpyruvate carboxylase (EC 4.1.1.31)	L3K52_06505	L2Y54_RS07775
pyc	Pyruvate carboxylase (EC 6.4.1.1)	L3K52_11710	L2Y54_RS14905
**Antioxidant protection**			
SOD2	Superoxide dismutase [Fe] (EC 1.15.1.1)	L3K52_00175	L2Y54_RS00165
CAT2	catalase/peroxidase HPI (EC 1.11.1.21)	L3K52_09360	L2Y54_RS11910
CAT	Catalase (EC 1.11.1.6)	L3K52_07200	-
ccp	Cytochrome c551 peroxidase (EC 1.11.1.5)	L3K52_05920<br>L3K52_09275	L2Y54_RS07160<br>L2Y54_RS11850<br>L2Y54_RS11925
**Respiratory Electron Transfer Chain**			
**Complex 1 NADH-dehydrogenase**			
nuoA	NADH ubiquinone oxidoreductase chain A (EC 1.6.5.3)	L3K52_08960	L2Y54_RS11595

nouB	NADH-ubiquinone oxidoreductase chain B (EC 1.6.5.3)	L3K52_08965	L2Y54_RS11600
nuoC	NADH-ubiquinone oxidoreductase chain C (EC 1.6.5.3)	L3K52_08970	L2Y54_RS11605
nuoD	NADH-ubiquinone oxidoreductase chain D (EC 1.6.5.3)	L3K52_08975	L2Y54_RS11610
nuoE	NADH-ubiquinone oxidoreductase chain E (EC 1.6.5.3)	L3K52_08980	L2Y54_RS11615
nuoF	NADH-ubiquinone oxidoreductase chain F (EC 1.6.5.3)	L3K52_08985	L2Y54_RS11620
nuoG	NADH-ubiquinone oxidoreductase chain G (EC 1.6.5.3)	L3K52_08990	L2Y54_RS11625
nuoH	NADH-ubiquinone oxidoreductase chain H (EC 1.6.5.3)	L3K52_08995	L2Y54_RS11630
nuoI	NADH-ubiquinone oxidoreductase chain I (EC 1.6.5.3)	L3K52_09000	L2Y54_RS11635
nuoJ	NADH-ubiquinone oxidoreductase chain J (EC 1.6.5.3)	L3K52_09005	L2Y54_RS11640
nuoK	NADH-ubiquinone oxidoreductase chain K (EC 1.6.5.3)	L3K52_09010	L2Y54_RS11645
nuoL	NADH-ubiquinone oxidoreductase chain L (EC 1.6.5.3)	L3K52_09015<br>L3K52_01510	L2Y54_RS11650
nuoM	NADH-ubiquinone oxidoreductase chain M (EC 1.6.5.3)	L3K52_09020<br>L3K52_01500<br>L3K52_01515<br>L3K52_01520	L2Y54_RS11655<br>L2Y54_RS00890<br>L2Y54_RS00915<br>L2Y54_RS00920
nuoN	NADH-ubiquinone oxidoreductase chain N (EC 1.6.5.3)	L3K52_09025	L2Y54_RS11660
**Complex II (succinate dehydrogenase)**			
sdhC	Succinate dehydrogenase, cytochrome b556 subunit (EC 1.3.99.1)	L3K52_13210	L2Y54_RS12470
sdhD	Succinate dehydrogenase, hydrophobic	L3K52_13215	L2Y54_RS12465

	membrane anchor protein (EC 1.3.5.1)		
sdhA	Succinate dehydrogenase flavoprotein subunit (EC 1.3.99.1)	L3K52_13200	L2Y54_RS12480
sdhB	Succinate dehydrogenase iron-sulfur protein (EC 1.3.99.1)	L3K52_13205	L2Y54_RS10030
**Complex III**			
cytB	ubiquinol-cytochrome c reductase cytochrome b subunit (EC 7.1.1.8)	L3K52_16505	L2Y54_RS19170
cyc1	ubiquinol-cytochrome c reductase cytochrome c1 subunit (EC 7.1.1.8)	L3K52_16500	L2Y54_RS19165
rip1 (ISP)	ubiquinol-cytochrome c reductase iron-sulfur subunit (EC 7.1.1.8)	L3K52_16510<br>L3K52_10880	L2Y54_RS15965<br>L2Y54_RS19175
**Complex IV (terminal oxydases)**			
cyoE	Heme A synthase, cytochrome oxidase biogenesis protein Cox15-CtaA (EC 2.5.1.141)	L3K52_00155	L2Y54_RS00145
cox11	cytochrome c oxidase assembly protein subunit 11 (EC 7.1.1.9)	L3K52_00125	L2Y54_RS00115
cox15	cytochrome c oxidase assembly protein subunit 15 (EC 7.1.1.9)	L3K52_00100<br>L3K52_00155	L2Y54_RS00085
coxB	cytochrome c oxidase subunit II (EC 7.1.1.9)	L3K52_00115	L2Y54_RS00100
coxA	cytochrome c oxidase subunit I (EC 7.1.1.9)	L3K52_00120	L2Y54_RS00105
coxC	cytochrome c oxidase subunit III (EC 7.1.1.9)	L3K52_00130	L2Y54_RS00120

coxD	cytochrome c oxidase subunit IV (EC 7.1.1.9)	-	L2Y54_RS20060
cydA	Cytochrome d ubiquinol oxidase subunit I (EC 7.1.1.7)	L3K52_15575	-
cydB	Cytochrome d ubiquinol oxidase subunit II (EC 7.1.1.7)	L3K52_15570	-
cydC	Transport ATP-binding protein CydC	L3K52_15555	-
cydD	Transport ATP-binding protein CydD	L3K52_15560	-
ccoN	cytochrome c oxidase cbb3-type subunit I (EC 1.9.3.1)	L3K52_14130	L2Y54_RS16310<br>L2Y54_RS19630
ccoO	cytochrome c oxidase cbb3-type subunit II (EC 7.1.1.9)	L3K52_14125	L2Y54_RS16305<br>L2Y54_RS19625
ccoP	cytochrome c oxidase cbb3-type subunit III (EC 7.1.1.9)	L3K52_14115	L2Y54_RS16295<br>L2Y54_RS19615
ccoG (FixG)	Type cbb3 cytochrome oxidase biogenesis protein CcoG, involved in Cu oxidation	L3K52_14635	L2Y54_RS19610<br>L2Y54_RS17630
ccoQ	CcoQ/FixQ family Cbb3-type cytochrome c oxidase assembly chaperone (EC 1.9.3.1)	-	L2Y54_RS19620
ccoS	cbb3-type cytochrome oxidase assembly protein CcoS	L3K52_14620	L2Y54_RS17615
**ATPase**			
atpA	F-type H+-transporting ATPase subunit alpha (EC 7.1.2.2)	L3K52_00430	L2Y54_RS00385<br>L2Y54_RS09295
atpD	F-type H+-transporting ATPase subunit beta (EC 7.1.2.2)	L3K52_00420	L2Y54_RS00375
atpH	F-type H+-transporting ATPase	L3K52_00435	L2Y54_RS00390

	subunit delta (EC 7.1.2.2)		
atpC	F-type H+-transporting ATPase subunit epsilon (EC 7.1.2.2)	L3K52_00415	L2Y54_RS00370
atpG	F-type H+-transporting ATPase subunit gamma (EC 7.1.2.2)	L3K52_00425	L2Y54_RS00380
atpB	F-type H+-transporting ATPase subunit a (EC 7.1.2.2)	L3K52_00450	L2Y54_RS00405
atpF	F-type H+-transporting ATPase subunit b (EC 7.1.2.2)	L3K52_00440	L2Y54_RS00395
atpE	ATP synthase F0 sector subunit c (EC 7.1.2.2)	L3K52_00445	L2Y54_RS00400
